# An optimized method for IgE-mediated degranulation of human lung mast cells

**DOI:** 10.3389/fimmu.2024.1393802

**Published:** 2024-05-31

**Authors:** Yitao Gong, Anna-Karin Johnsson, Jesper Säfholm, Mamdoh Al-Ameri, Erik Sachs, Kasra Vali, Gunnar Nilsson, Elin Rönnberg

**Affiliations:** ^1^ Division of Immunology and Allergy, Department of Medicine Solna, and Center for Molecular Medicine, Karolinska Institutet, and Karolinska University Hospital, Stockholm, Sweden; ^2^ Unit of Integrative Metabolomics, Institute of Environmental Medicine, Karolinska Institutet, Stockholm, Sweden; ^3^ Department of Respiratory Medicine and Allergy, Karolinska University Hospital, Stockholm, Sweden; ^4^ Department of Molecular Medicine and Surgery, Karolinska Institutet, Stockholm, Sweden; ^5^ Department of Cardiothoracic Surgery, Karolinska University Hospital, Stockholm, Sweden; ^6^ Department of Medical Sciences, Uppsala University, Uppsala, Sweden

**Keywords:** human lung mast cells, IgE-mediated degranulation, FcεRI, degranulation, MRGPRX2

## Abstract

**Background:**

Mast cells are critically involved in IgE-mediated diseases, e.g., allergies and asthma. Human mast cells are heterogeneous, and mast cells from different anatomical sites have been shown to respond differently to certain stimuli and drugs. The origin of the mast cells is therefore of importance when setting up a model system, and human lung mast cells are highly relevant cells to study in the context of asthma. We therefore set out to optimize a protocol of IgE-mediated activation of human lung mast cells.

**Methods:**

Human lung mast cells were extracted from lung tissue obtained from patients undergoing pulmonary resection by enzyme digestion and mechanical disruption followed by CD117 magnetic-activated cell sorting (MACS) enrichment. Different culturing media and conditions for the IgE-mediated degranulation were tested to obtain an optimized method.

**Results:**

IgE crosslinking of human lung mast cells cultured in serum-free media gave a stronger response compared to cells cultured with 10% serum. The addition of stem cell factor (SCF) did not enhance the degranulation. However, when the cells were put in fresh serum-free media 30 minutes prior to the addition of anti-IgE antibodies, the cells responded more vigorously. Maximum degranulation was reached 10 minutes after the addition of anti-IgE. Both CD63 and CD164 were identified as stable markers for the detection of degranulated mast cells over time, while the staining with anti-CD107a and avidin started to decline 10 minutes after activation. The levels of CD203c and CD13 did not change in activated cells and therefore cannot be used as degranulation markers of human lung mast cells.

**Conclusions:**

For an optimal degranulation response, human lung mast cells should be cultured and activated in serum-free media. With this method, a very strong and consistent degranulation response with a low donor-to-donor variation is obtained. Therefore, this model is useful for further investigations of IgE-mediated mast cell activation and exploring drugs that target human lung mast cells, for instance, in the context of asthma.

## Introduction

Mast cells are granulated cells that play a major role in allergic diseases and asthma. The allergic activation of mast cells occurs by crosslinking of allergen-specific IgE antibodies that are bound to the high-affinity IgE receptor, FcεRI. The mast cells respond to this activation by releasing their pre-formed granule content, including histamine, mast cell proteases (tryptase and chymase carboxypeptidase A3), and heparin, and by *de novo* synthesizing lipid mediators (prostaglandin D_2_, cysteine leukotrienes, etc.) and cytokines. These mediators will in turn cause the allergic symptoms, such as smooth muscle cell constriction, mucus production, edema, and itch (1).

Mast cells have been shown to be heterogeneous, and human mast cells are divided into the MC_T_ and MC_TC_ subtypes, depending on their expression of mast cell proteases. MC_T_ expresses tryptase and MC_TC_, in addition to tryptase, also express chymase, carboxypeptidase A3, and cathepsin G (2–4). The prevalence of these subtypes varies in various tissues, where, for example, mast cells in the skin are predominantly of the MC_TC_ type, and in the lung, they are predominantly of the MC_T_ type (2). Mast cells from the skin have also been shown to express MRGPRX2, a receptor that binds numerous basic compounds, such as compound 48/80, substance P, LL-37, and various drugs, causing degranulation and pseudo-allergic reactions (5–7). Lung mast cells of the MC_T_ type, in contrast, lack the expression of this receptor and therefore do not respond to these stimuli (8, 9). In addition, it is known that mast cells from different tissues have different levels of sensitivity to drugs. For example, lung but not skin mast cells are inhibited by sodium cromoglycate (10). Therefore, the origin of the mast cells is important when setting up a model system and testing the effect of potential activators and drugs on human mast cells. Thus, using mast cells obtained from human lungs is a highly relevant model for studying IgE-mediated degranulation in the context of allergic asthma.

We previously developed a method to extract mast cells from human lung tissue with high yield (11). These human lung mast cells can be used to study degranulation in different contexts and the potential effect of various drugs on the reaction. However, we and others found very high donor-to-donor and experiment-to-experiment variations in the degranulation response, with some donors showing very limited activation while others show a very strong response, and a minor change of the conditions will make a significant difference in terms of activation (12–15). We therefore set out to optimize the conditions in the experiments to obtain a high and stable degranulation response of human lung mast cells.

## Materials and methods

### Ethical approval

The study was approved by the local ethics committee (Regionala Etikprövningsnämnden Stockholm, 2018/1819–31/1), and all patients gave their informed consent for the collection of lung tissue during pulmonary resection procedures due to cancer. Lung tissue distal to the tumor was utilized.

### Cell preparation

Human lung mast cells were obtained from lung tissues as essentially described in a previous study (11). In brief, human lung tissue was cut into small pieces and enzymatically digested for 30 minutes with 0.2 mg/mL DNase I (Sigma-Aldrich, St. Louis, MO, USA) and 0.1 mg/mL Liberase TL (Sigma-Aldrich). Thereafter, the tissue was vigorously cut by scissors and mechanically disrupted by plunging through a syringe. The cells were purified by 30% Percoll centrifugation, and red blood cells were lysed using ACK lysis buffer (Thermo Fisher, Waltham, MA, USA). The cells were then cultured in StemPro™-34 SFM media (Gibco, Grand Island, NY, USA) [StemPro™-34 SFM media include the supplied nutrient supplement and 2 mM l-glutamine (Sigma-Aldrich) unless otherwise stated] overnight, dead cells were thereafter removed by Dead Cell Removal Kit (Miltenyi, Bergisch Gladbach, Germany), and mast cells were enriched by magnetic-activated cell sorting (MACS) enrichment using CD117 MicroBead Kit (Miltenyi). Enriched cells were cultured in StemPro™-34 SFM media with 2 mM l-glutamine or in RPMI 1640 media (HyClone-Cytiva) with 10% fetal bovine serum (FBS; Sigma-Aldrich). Both media were supplemented with 1% penicillin/streptomycin (Sigma) and 100 ng/mL stem cell factor (SCF) (Sobi, Stockholm, Sweden). The cells were cultured for at least 4 days before IgE-crosslinking experiments were performed.

### IgE crosslinking

One day prior to crosslinking, 1 μg/mL of human IgE (Myeloma, Calbiochem, San Diego, CA, USA) was added to the media. After washing away unbound IgE, the cells were crosslinked with various concentrations of anti-IgE (clone GE-1, Sigma-Aldrich) for different time periods at 37°C in StemPro™-34 SFM media or PIPES-BSA buffer [0.2% bovine serum albumin (BSA) (Sigma-Aldrich), 9.3 mM PIPES sodium salt (Sigma-Aldrich), 140.0 mM sodium acetate trihydrate, 5.0 mM potassium acetate, 0.6 mM calcium chloride dihydrate, and 0.11 mM magnesium chloride hexahydrate, pH adjusted to 7.4 with 1 M Tris]. After activation, cells were washed with phosphate-buffered saline (PBS) + 2% FBS and thereafter stained with different antibodies and analyzed by flow cytometry.

### MRGPRX2-mediated activation

The cells were plated and allowed to pre-incubate in fresh StemPro™-34 SFM media for 30 minutes prior to the addition of 10 µg/mL compound 48/80 (Sigma-Aldrich). After 10 minutes, the cells were washed with PBS + 2% FBS and thereafter stained with antibodies and analyzed by flow cytometry.

### Flow cytometry

The following antibodies/stains were used: anti-CD117-APC (clone 104D2, BD Biosciences, San Jose, CA, USA), anti-CD107a-APC-H7 (clone H4A3, BD Biosciences), anti-CD164-PE (clone 67D2, BioLegend, San Diego, CA, USA), anti-CD63-FITC (clone H5C6, BD Biosciences), anti-CD63-PE-Cy7 (clone H5C6, BD Biosciences), anti-CD13-BV421 (clone WM15, BioLegend), anti-CD203c-FITC (clone NP4D6, BioLegend), and Avidin-Alexa Fluor 488 (Thermo Fisher). Staining was performed by incubation for at least 8 minutes at 4°C with the antibodies 1:100 diluted in PBS + 2% FBS, followed by washing with PBS + 2% FBS. The cells were resuspended in PBS + 2% FBS, and DAPI (BD Biosciences) was added to a final dilution of 1:10,000 prior to running the flow cytometry on a BD FACSCanto (BD Biosciences). Live Human lung mast cells (HLMCs) were gated as DAPI negative and CD117 high. Gates for positive degranulation were set according to the anti-CD63 (or other markers when they were used) stained non-activated control. Flow cytometry data analysis was conducted using FlowJo software version 10.8.1 (FlowJo LLC, Ashland, OR, USA).

### Statistical analysis

Student’s two-tailed t-test was used to compare differences between the two groups; the Mann–Whitney test was performed when the normality test (α = 0.05) was not passed. One-way ANOVA or two-way ANOVA tests with Bonferroni correction for multiple comparisons were performed when comparing more than two groups. Statistical analyses were performed using GraphPad Prism software version 9.4.1. * p < 0.05; ** p < 0.01; *** p < 0.001; **** p < 0.0001.

### Stepwise protocol for optimized human lung mast cell degranulation

#### Materials

StemPro™-34 SFM media (Gibco) [with the supplied nutrient supplement and 2 mM l-glutamine (Sigma-Aldrich)] or RPMI 1640 media (HyClone-Cytiva)
Cell culture flask (T-25, surface: suspension, filter cap, SARSTEDT, Nümbrecht Germany)
96-well cell culture plate (surface: suspension, bottom shape: conical, SARSTEDT)
SCF (Sobi)
Human IgE, Myeloma (Calbiochem)
Anti-IgE, clone GE-1 (Sigma-Aldrich)
Fluorescence-activated cell sorting (FACS) buffer (PBS + 2% FBS)
Anti-CD63-PE-Cy7 (clone H5C6, BD Biosciences)
Anti-CD117-APC (clone 104D2, BD Biosciences)
DAPI (BD Biosciences)
BD FACSCanto (BD Biosciences or similar flow cytometer)

#### Stepwise protocol

1. Culture MACS-enriched human lung mast cells with StemPro™-34 SFM media with 100 ng/mL SCF at a concentration of 0.5 × 10^6^ cells/mL in cell culture flasks for at least 4 days.
2. Add 1 μg/mL of human IgE to the media 1 day prior to crosslinking and incubate in the cell incubator for one night.
3. Before IgE crosslinking, pre-heat StemPro™-34 SFM media (or serum-free RPMI) with and without 4 μg/mL anti-IgE antibodies to 37°C.
4. Collect cells from the flask and count the cell number.
5. Spin down the cells at 400 ×*g* for 5 minutes at room temperature and discard the supernatant.
6. Resuspend the cells to the concentration of 10^4^ cells/mL using pre-heated StemPro™-34 SFM media (or serum-free RPMI media).
7. Add 50 μL of cells per well (5,000 cells per well) into a conical bottom 96-well cell culture plate.
8. Pre-incubate at 37°C for 30 minutes.
9. Add 50 μL pre-heated 4 μg/mL anti-IgE to wells to make the final concentration 2 μg/mL or 50 μL pre-heated media to the control sample.
10. Incubate at 37°C for 10 minutes (optimal degranulation is reached at 10 minutes, but this time can be increased to investigate other effects).
11. Spin down the cells at 400 ×*g* for 5 minutes at 4°C. Optional: If the supernatant is collected, transfer the supernatant and store it at −20°C for later analysis of released mediators; otherwise, discard the supernatant.
12. Add 200 μL FACS buffer per well, spin down the cells at 400 ×*g* for 5 minutes at 4°C (maximum break), and discard the supernatant.
13. Resuspend with 50 μL per well 1:100 diluted anti-CD63 and anti-CD117 antibodies in FACS buffer and stain for 8 minutes at 4°C (maximum staining is reached at 8 minutes, but this time can also be increased).
14. Spin down the cells at 400 ×*g* for 5 minutes at 4°C (maximum break) and resuspend the cell pellet with 100 μL FACS buffer per well.
15. Prior to running the samples on the flow cytometer, add 100 μL per well of DAPI 1:5,000 diluted in FACS buffer.
16. Gate mast cells as live (DAPI low) and CD117 high and set a cutoff for degranulated cells according to the anti-CD63 stained non-stimulated control.

## Results

### HLMC degranulation is increased when cultured in serum-free StemPro™-34 SFM media

**Figure 1:**
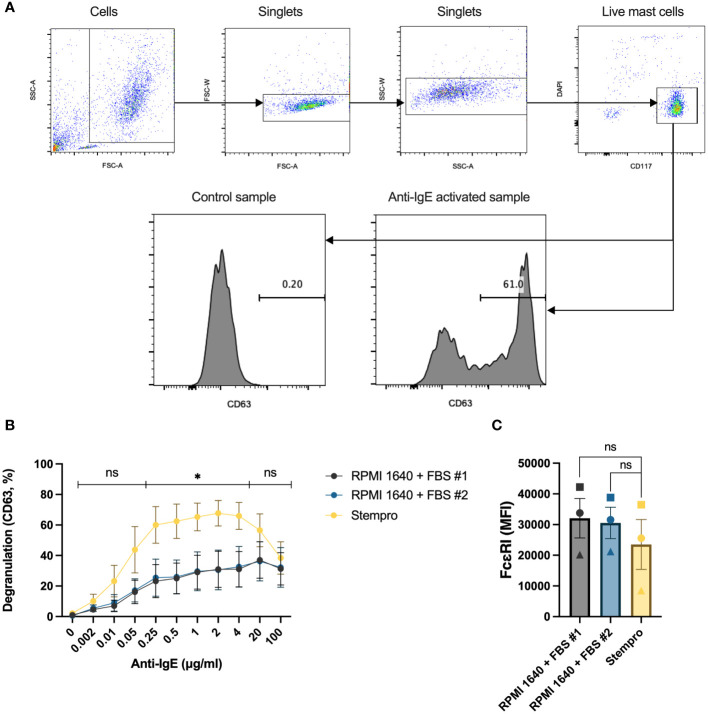
The effect of different culture media on HLMC degranulation. **(A)** Gating strategies for the degranulation experiments and representative CD63 histograms for control and anti-IgE activated samples. **(B)** HLMCs were cultured with serum-free StemPro™-34 SFM media and RPMI 1640 media supplemented with two different batches of FBS for 4 days before they were crosslinked with anti-IgE in PIPES-BSA media. Degranulation was evaluated by flow cytometric detection of anti-CD63 staining. n = 5; means with SEMs. **(C)** The FcϵRI expression [median fluorescence intensity (MFI)] in the different treatment groups was detected by flow cytometry; each individual donor is represented by a unique symbol. n = 3; means with SEMs. FBS, fetal bovine serum. ns, non significant. *p < 0.05.

Mast cells were extracted from human lung tissue using enzymatic and mechanical disruption of the tissue followed by CD117 MACS enrichment. The cells failed to degranulate in response to IgE-crosslinking directly after the preparation, they needed to rest for a couple of days to be able to respond (data not shown), they needed to rest for a couple of days to be able to respond. To investigate the effect of different cell culture media on HLMC degranulation, the cells were cultured for 4 days with serum-free StemPro™-34 SFM media or RPMI 1640 media with different FBS batches. Although we optimized the extraction of human lung mast cells from the tissue (11), the obtained number of these primary cells is limited. We therefore used flow cytometry with surface CD63 staining as a surrogate marker for degranulation to be able to assess degranulation at the single cell level and determine the degranulation percentage with relatively few cells utilized. The gating strategies and representative CD63 histograms are shown in **Figure 1A**. The degranulation peaked at 2 μg/mL anti-IgE antibodies, and cells cultured in StemPro™-34 SFM media had significantly higher degranulation percentages than cells cultured in RPMI 1640 with either of the two FBS batches (**Figure 1B**). Despite having different degranulation percentages, the high-affinity IgE receptor (FcϵRI) expression was similar in the different culturing conditions (**Figure 1C**). Because of this, we chose to culture the cells in StemPro™-34 SFM media and use 2 μg/mL anti-IgE antibodies for the rest of the experiments.

### HLMC degranulation response is higher in StemPro™-34 SFM media than in PIPES-BSA buffer

**Figure 2:**
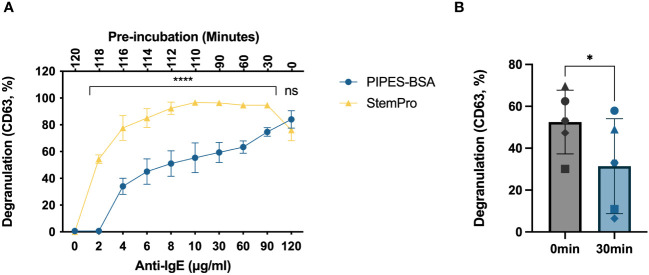
Degranulation time course of HLMCs crosslinked in different buffers. **(A)** The media were changed to either PIPES-BSA buffer or StemPro™-34 SFM media, the cells were plated on a 96-well plate, and 2 μg/mL anti-IgE was added immediately to the 120-minute timepoint; thereafter, anti-IgE was added at different timepoint to ensure that the cells had been incubated with anti-IgE for the indicated periods (lower x-axis) of time at the endpoint of the experiment. This also leads to the cells being pre-incubated in the media for various timepoints prior to the addition of the anti-IgE (upper x-axis). **(B)** Anti-IgE was added immediately after the media change or after 30-minute pre-incubation in PIPES-BSA media, and the reaction was stopped after 10 minutes; each individual donor is represented by a unique symbol. Degranulation was evaluated by flow cytometric detection of anti-CD63 staining. n = 3–5; means with SEMs. ns, non significant. *p < 0.05; ****p < 0.0001.

The media that HLMCs are IgE-crosslinked in, i.e., the degranulation media, also affect their response. We traditionally degranulated mast cells in a PIPES buffer with 2% BSA; however, we have not investigated how this buffer affects mast cell degranulation in time-course experiments where the cells are kept in the degranulation buffer for a longer period. We therefore compared the degranulation percentage when the cells were activated in the PIPES-BSA buffer or in StemPro™-34 SFM media, the media in which the HLMCs were cultured. To make this time course experimentally feasible, the media were changed to the degranulation media, i.e., PIPES-BSA or StemPro™-34 SFM media, the cells were plated, and the anti-IgE antibodies were added at different timepoints so that the endpoint and staining of the cells with anti-CD63 antibodies were performed at the same time (**Figure 2A**, lower x-axis). This means that the cells had been sitting in the degranulation media for different periods of time prior to the addition of the anti-IgE antibodies (**Figure 2A**, upper x-axis). At the 120-minute timepoint, when the anti-IgE was added immediately after the media change, there was no difference in degranulation response between the media (**Figure 2A**). However, at the timepoint of 90 minutes down to 2 minutes of activation, cells crosslinked in StemPro™-34 SFM media had a substantially higher degranulation than those in the PIPES-BSA buffer (**Figure 2A**). PIPES media only contain basic salts, and since the degranulation response was the highest for this buffer when the anti-IgE was added directly after the media change (120 timepoint), we hypothesized that cells that have been incubated for some time in this media may show lower degranulation. To test this, we performed IgE crosslinking for 10 minutes on cells pre-incubated in PIPES-BSA for 30 minutes or immediately after the media change, and the pre-incubated cells did indeed show lower degranulation (**Figure 2B**). Thus, the late peak in degranulation in the PIPES buffer is not due to a slow degranulation but rather a lowering in the degranulation response by pre-incubation in this media prior to IgE crosslinking (**Figure 2A**). The degranulation response reached maximum in the StemPro™-34 SFM media at 10 minutes and was stable up until 90 minutes (**Figure 2A**). Therefore, henceforth, we used StemPro™-34 SFM media as degranulation media.

### CD63 is a stable marker for HLMC degranulation

**Figure 3:**
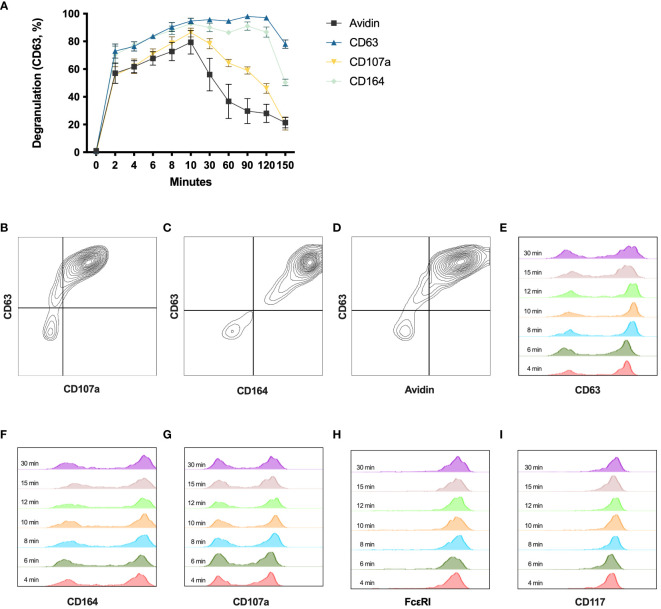
Degranulation time course with different degranulation markers. **(A)** HLMCs were incubated with 2 μg/mL anti-IgE from 0 to 150 minutes before being stained with avidin, anti-CD63, anti-CD107a, or anti-CD164 and analyzed by flow cytometry. n = 3; means with SEMs. **(B–D)** Co-staining of indicated markers at 10 minutes after IgE crosslinking is shown. **(E–I)** Anti-IgE crosslinked HLMCs (10 minutes) were stained with indicated markers for different timepoints (indicated in the figure) on ice before being washed and analyzed on the flow cytometer.

In addition to CD63 (16), CD107a, CD164, CD203c, CD13 (17), and fluorochrome-labeled avidin (18, 19) can also be used to evaluate degranulation by flow cytometry. To test the stability of these markers for degranulation over time, HLMCs were stained after being crosslinked with 2 μg/mL anti-IgE at 37°C for different time periods. The surface expression of CD203c and CD13 did not increase in degranulated HLMCs (data not shown). In contrast, CD63, CD107a, CD164, and avidin performed similarly and reached their maximum 10 minutes after the addition of anti-IgE (**Figure 3A**). These markers also correlated well with each other, staining the same degranulated mast cells at the 10 minutes timepoint (**Figures 3B–D**). After 10 minutes, avidin and CD107a started to decline, indicating that these markers were not stable over time. In contrast, CD63 and CD164 were stable up until 120 minutes (**Figure 3A**). We also investigated the optimal staining time for the detection of the different surface markers and found that staining the cells for 8 minutes was enough to reach maximum signal and clear distinction of degranulated and non-degranulated mast cells (**Figures 3E–I**).

### Change of media and addition of SCF have no significant effect on HLMC degranulation

**Figure 4:**
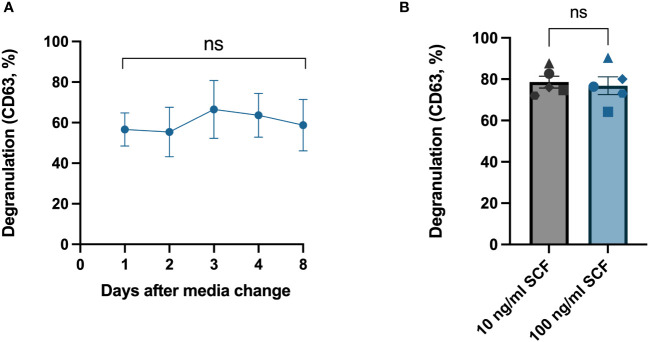
Change of media and addition of fresh SCF had no significant effect on HLMC degranulation. **(A)** IgE crosslinking was performed on different days after media change to fresh StemPro™-34 SFM media and 100 ng/mL SCF. n = 4; means with SEMs. **(B)** HLMCs were cultured in StemPro™-34 SFM media with different concentrations of SCF for 1 day before IgE crosslinking; each individual donor is represented by a unique symbol. n = 5; means with SEMs. Degranulation was evaluated by flow cytometric detection of anti-CD63 staining. SCF, stem cell factor. ns, non significant.

SCF is a pivotal growth factor for human mast cells that they require for their survival. Additionally, it is reported that SCF also enhances mast cell degranulation (15). We therefore next investigated if the degranulation response would differ if it was performed at different times in relation to media change and the addition of SCF. We hence tested the degranulation response on different days after the media change. We replaced the media of the HLMCs every 7–8 days, and the degranulation response was stable throughout this period (**Figure 4A**); therefore, it does not matter when degranulation experiments are performed in relation to media change. We also cultured HLMCs in different SCF concentrations (10 ng/mL and 100 ng/mL) in StemPro™-34 SFM media for 1 day and found no significant difference between the two groups (**Figure 4B**).

### Pre-incubation in fresh StemPro™-34 SFM media before crosslinking significantly increases HLMC degranulation

**Figure 5:**
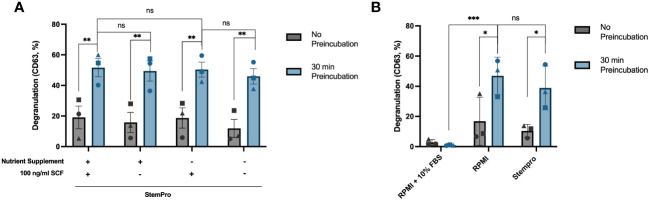
Pre-incubation in fresh StemPro™-34 SFM media increases HLMC degranulation. **(A)** HLMCs were pre-incubated in fresh StemPro™-34 SFM media with or without SCF/nutrient supplement for 30 minutes at 37°C before IgE crosslinking, or the degranulation was performed immediately after the media change (no pre-incubation). **(B)** HLMCs were pre-incubated in different media, RPMI 1640 media with 10% serum, RPMI alone, or StemPro™-34 SFM media (with nutrient supplement), for 30 minutes at 37°C before IgE crosslinking, or the degranulation was performed immediately after the media change (no pre-incubation). Degranulation was evaluated by flow cytometric detection of anti-CD63 staining. Each individual donor is represented by a unique symbol, n = 3; means with SEMs. SCF, stem cell factor; RPMI, RPMI-1640 media. ns, non significant. *p < 0.05; **p < 0.01; *** p < 0.001.

We noticed in our time course experiments that the degranulation response dropped at the last timepoint (**Figures 2A**, **3A**). Since in the latest timepoint the anti-IgE antibodies were added immediately after the media change while in the other timepoints the cells had been incubated in the media for various timepoints (**Figure 2A**, upper x-axis), we decided to test whether pre-incubating in fresh media could have an impact on the response. We found that the degranulation response increased significantly when the cells were allowed to be pre-incubated in fresh StemPro™-34 SFM media for 30 minutes before activation, compared to cells activated immediately after the media change (**Figure 5A**). In other words, giving the cells fresh StemPro™-34 SFM media primed HLMCs for degranulation. To exclude that the presence of SCF in the StemPro™-34 SFM media was the cause of the effect, we compared StemPro™-34 SFM media with or without SCF, and with or without the nutrient supplement that was supplied with StemPro™-34 SFM media (and normally always added to the StemPro™-34 SFM media), and the results were comparable (**Figure 5A**). We also tested to pre-incubate the cells in RPMI media (without serum added) for 30 minutes prior to degranulation, and also in this case, the response was increased to a similar extent as in StemPro™-34 SFM media. However, when cells were pre-incubated in RPMI media with 10% serum, the degranulation was significantly reduced (**Figure 5B**).

### Human lung mast cells do not respond to the MRGPRX2 agonist compound 48/80

**Figure 6:**
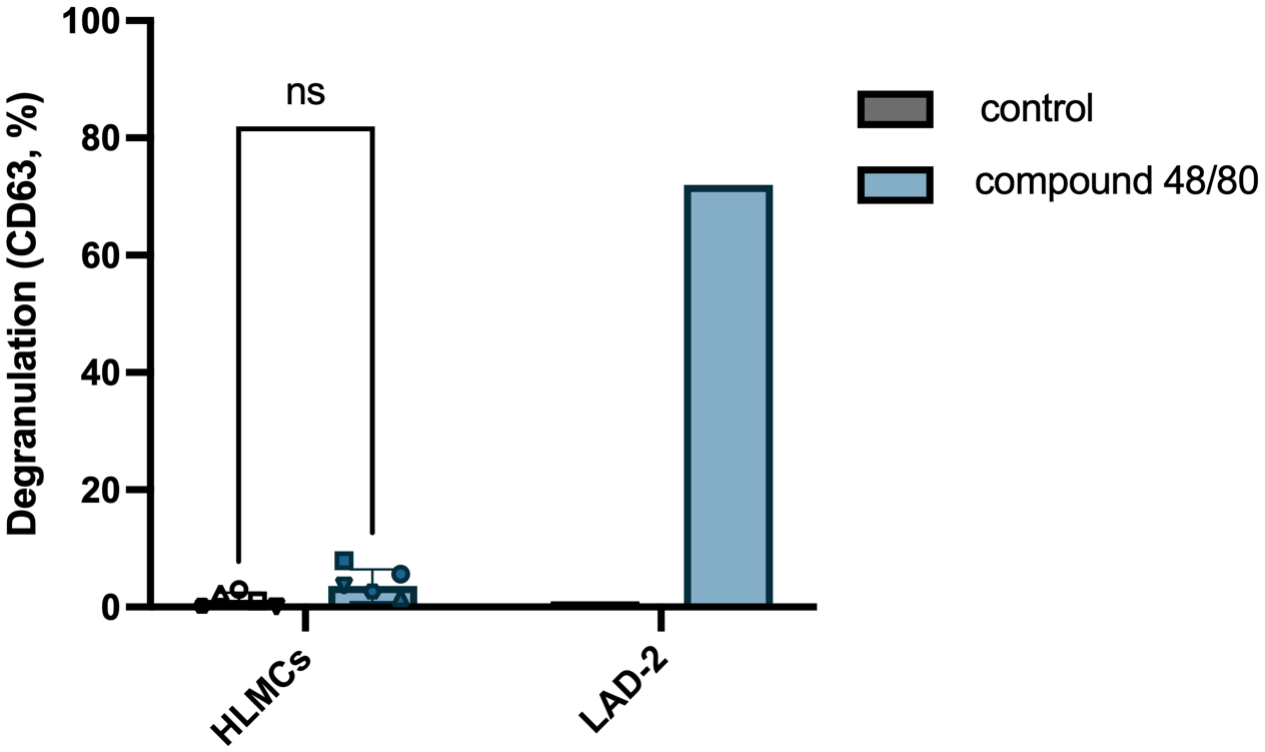
HLMCs do not degranulate in response to MRGPRX2 agonist compound 48/80. HLMCs were pre-incubated in fresh StemPro™-34 SFM media for 30 minutes prior to addition of 10 µg/mL compound 48/80. LAD-2 cells were used as positive control for MRGPRX2-induced degranulation. Degranulation was evaluated by flow cytometric detection of anti-CD63 staining. Each individual donor is represented by a unique symbol, n = 6; means with SEMs. ns, non significant.

It has been published that HLMCs do not express the MRGPRX2 receptor and that they do not degranulate upon exposure to MRGPRX2 agonists (8, 9). We tested whether this is also true using our optimized culturing/degranulation method with MRGPRX2 agonist compound 48/80. In line with previously published results, we found that compound 48/80 does not induce significant degranulation in HLMCs, while the mast cell line LAD-2, in contrast, readily degranulates upon exposure to compound 48/80 (**Figure 6**).

## Discussion

Because of a high variation in the IgE-mediated degranulation response that we have observed in HLMCs, we set out to optimize the conditions to obtain a higher, more stable, and reproducible response by the IgE crosslinking of the HLMCs.

HLMCs fail to degranulate when the IgE crosslinking is performed directly after extraction of the cells from the lung tissue (data not shown). The extraction and purification protocols are very harsh on the cells, and it is likely that the cells are activated in this process. This could be a reason why they cannot degranulate in response to the IgE crosslinking; however, it is not possible to control for this since we need to extract the cells to perform the experiment. Regardless of the reason, the cells need to rest for a few days before they can respond to IgE crosslinking by degranulation. We found that culturing the HLMCs in a serum-free media results in increased IgE-mediated degranulation compared to cells cultured in RPMI with 10% serum. There was no statistical difference in the surface expression of the FcεRI receptor; thus, the receptor expression is not likely to be the cause of the lower response.

SCF has been reported to prime mast cells and increase the degranulation of the cells upon IgE crosslinking (20), also specifically in HLMCs (15). In our experiments, SCF did not influence the degree of degranulation (**Figures 4A, B**, **5A**). The rationale for this inconsistency remains speculative. One possibility is that the level of degranulation is already maximized under our optimized conditions, leaving no scope for any additional priming effect of SCF.

We found that merely changing the media of the cells, i.e., placing them in fresh StemPro™-34 SFM 30 minutes before activation, increased the rate of degranulation (**Figure 5A**), which suggests that there may be some ingredients in StemPro™-34 SFM media that prime mast cells for degranulation, or there is an inhibitory factor in the culture media that is removed. StemPro™-34 SFM media are a trademark serum-free media, consisting of a basal liquid medium and a frozen nutrient supplement. Initially, we hypothesized that the nutrient supplement contained ingredients that primed the mast cells; however, using only the basal liquid media without the supplement had the same priming effect (**Figure 5A**). In addition, we found that pre-incubating the cells in fresh RPMI media without serum added also had the same priming effect (**Figure 5B**). Pre-incubation in serum-containing RPMI 1640 media in contrast significantly reduced degranulation, suggesting inhibitory factor(s) present in the serum. It has previously been published that a factor in serum can inhibit mast cell degranulation (21); however, the identity of this factor is unknown.

Several different markers have been used to identify degranulated cells, including CD63, CD13, CD203c, CD107a, CD164, and avidin (which binds electrostatically to the exteriorized proteoglycans) (17–19). To measure the dynamics of these degranulation markers over time on the HLMCs, we set up a time course experiment, staining the mast cells at different timepoints after the IgE crosslinking. CD203c and CD13 staining did not increase in degranulated HLMCs (data not shown), while the staining for all the other markers increased and reached maximum 10 minutes after the activation (**Figure 3A**). Furthermore, the surface expression of the different markers also correlated strongly with each other (**Figures 3B–D**), showing that the markers identify the same degranulated mast cells. CD63 and CD164 were quite stable over time, while the CD107a and avidin staining started to drop earlier (**Figure 3A**). This is in contrast to a study on basophils where the CD203c, CD164, and CD13 reached maximum staining within 5–15 minutes, while CD63 and CD107a reached maximum after only 20–40 minutes (17), indicating that the degranulation process somewhat differs in basophils and mast cells. In summary, the CD63 staining was the marker that showed the highest degranulation percentage with a clear distinction between degranulated and non-degranulated cells, and it was stable over time; therefore, we believe that this is a useful marker for studying the degranulation of HLMCs.

In summary, our optimized method for degranulation of HLMCs consists of culturing the HLMCs in serum-free StemPro™-34 SFM media and pre-incubating the cells for 30 minutes in fresh StemPro™-34 SFM media (or serum-free RPMI) prior to activation by 2 μg/mL anti-IgE. This leads to peak degranulation at 10 minutes, which can be measured through flow cytometric analysis of CD63.

This optimized method consistently yields a high and stable activation percentage, proving valuable for the analysis of IgE-mediated activation in HLMCs and for assessing the impact of drugs targeting HLMCs in the context of lung diseases, such as asthma.

## Data availability statement

The original contributions presented in the study are included in the article/supplementary material. Further inquiries can be directed to the corresponding author.

## Ethics statement

The studies involving humans were approved by Regionala etikprövningsnämnden i Stockholm. The studies were conducted in accordance with the local legislation and institutional requirements. The participants provided their written informed consent to participate in this study.

## Author contributions

YG: Conceptualization, Data curation, Formal analysis, Investigation, Methodology, Software, Validation, Visualization, Writing – original draft, Writing – review & editing. AJ: Investigation, Methodology, Resources, Writing – review & editing. JS: Funding acquisition, Project administration, Resources, Writing – review & editing. ES: Resources, Writing – review & editing. MA: Resources, Writing – review & editing. KV: Resources, Writing – review & editing. GN: Conceptualization, Funding acquisition, Project administration, Supervision, Writing – review & editing. ER: Conceptualization, Data curation, Formal analysis, Funding acquisition, Investigation, Methodology, Project administration, Resources, Software, Supervision, Validation, Visualization, Writing – original draft, Writing – review & editing.
